# Single-center initial experience with inner-branch complex EVAR in 44 patients

**DOI:** 10.3389/fcvm.2023.1188501

**Published:** 2023-06-15

**Authors:** Marvin Kapalla, Albert Busch, Brigitta Lutz, Heiner Nebelung, Steffen Wolk, Christian Reeps

**Affiliations:** ^1^ Department of Visceral, Thoracic and Vascular Surgery, University Hospital Carl Gustav Carus Dresden at the Technical University Dresden, Dresden, Germany; ^2^ Institute and Polyclinic for Diagnostic and Interventional Radiology, University Hospital Carl Gustav Carus Dresden at the Technical University Dresden, Dresden, Germany

**Keywords:** complex endovascular aortic repair, inner branches, thoracoabdominal aneurysm repair, off-the-shelf, aortic stent graft

## Abstract

**Purpose:**

The use of inner-branch aortic stent grafts in the treatment of complex aortic pathologies aims at broad applicability and stable bridging stent sealing compared to other endovascular technologies. The objective of this study was to evaluate the early outcomes with a single manufacturer custom-made and off-the-shelf inner-branched endograft in a mixed patient cohort.

**Methods:**

This retrospective, monocentric study between 2019 and 2022 included 44 patients treated with inner-branched aortic stent grafts (iBEVAR) as custom-made device (CMD) or off-the-shelf device (E-nside) with at least four inner branches. The primary endpoints were technical and clinical success.

**Results:**

Overall, 77% (*n* = 34) and 23% (*n* = 10) of the patients (mean age 77 ± 6.5 years, *n* = 36 male) were treated with a custom-made iBEVAR with at least four inner branches and an off-the-shelf graft, respectively. Treatment indications were thoracoabdominal pathologies in 52.2% (*n* = 23), complex abdominal aneurysms in 25% (*n* = 11), and type Ia endoleaks in 22.7% (*n* = 10). Preoperative spinal catheter placement was performed in 27% (*n* = 12) of patients. Implantation was entirely percutaneous in 75% (*n* = 33). Technical success was 100%. Target vessel success manifested at 99% (178/180). There was no in-hospital mortality. Permanent paraplegia developed in 6.8% (*n* = 3) of patients. The mean follow-up was 12 months (range 0–52 months). Three late deaths (6.8%) occurred, one related to an aortic graft infection. Kaplan–Meier estimated 1-year survival manifested at 95% and branch patency at 98% (177/180). Re-intervention was necessary for a total of six patients (13.6%).

**Conclusions:**

Inner-branch aortic stent grafts provide a feasible option for the treatment of complex aortic pathologies, both elective (custom-made) and urgent (off-the-shelf). The technical success rate is high with acceptable short-term outcomes and moderate re-intervention rates comparable to existing platforms. Further follow-up will evaluate long-term outcomes.

## Introduction

Thoracoabdominal aortic aneurysms (TAAAs) are among the most challenging cases for vascular surgeons and remain considerable even since the implementation of complex endovascular treatment perioperative morbidity and mortality (1). Fenestrated and branched endografts (f/bEVAR) have reduced perioperative mortality and morbidity considerably, yet the ideal endovascular solution regarding specific complications, such as endoleaks, bridging stent occlusion, and migration remains controversial (2, 3). So far, patient-specific, custom-made endografts with fenestrations or outer branches have been implemented widely for the elective and acute setting with technical success rates of approximately 100%. Yet, the complication rates of 6%–10% spinal cord ischemia (SCI), 15% renal deterioration, and re-intervention rates up to 25% during the first 12 months have to be noticed and should be discussed with the patients (4–10).

**Figure 1:**
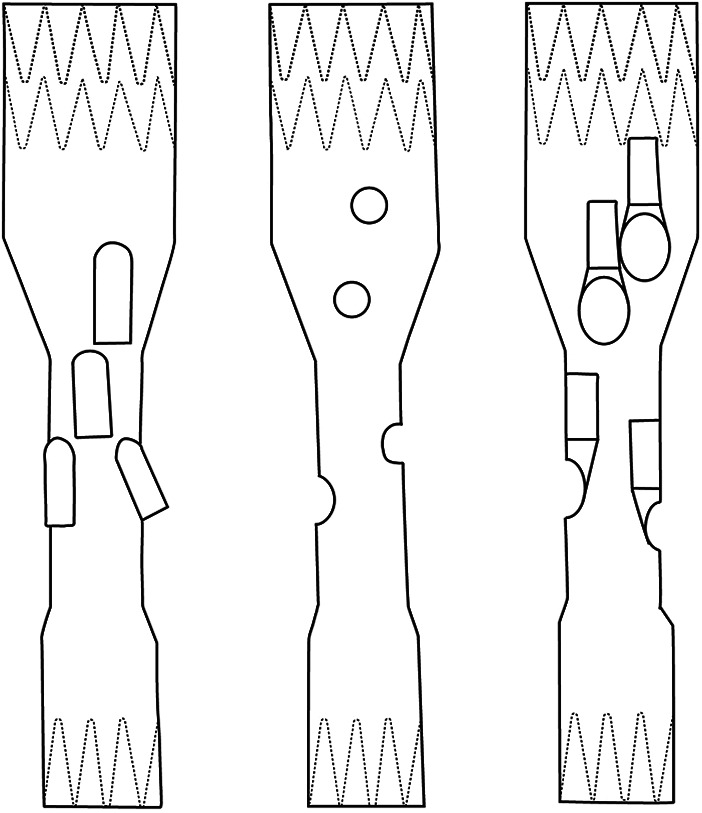
Sketches of FEVAR, BEVAR, and iBEVAR. Left: BEVAR (outer branches). Middle: FEVAR (fenestrations). Right: iBEVAR (inner branches).

While branched technology has demonstrated better long-term results regarding patency and prosthesis integrity, typically a narrow visceral aortic segment is still an indication for fenestrated grafts (6, 7, 10–15). Here, the latest configuration available, the inner-branched EVAR (iBEVAR), aimed to overcome these potential limitations (Figure 1). Advantages include increased anatomical suitability in narrow aortas while providing enhanced sealing between the main body and the bridging stents (11, 13).

Recently, a pre-cannulated “off-the-shelf” endograft with four inner branches (E-nside; Artivion, Germany) has become available and enabling iBEVAR solution even for urgent and emergency cases (16, 17). Up to now, only 84 cases of iBEVAR procedures ≥4 inner branches have been reported in three small cohort studies (11, 13, 14).

Thus, this study aims to evaluate the initial experience with the custom-made and off-the-shelf inner-branched devices in complex aortic aneurysm repair in a mixed cohort from a high-volume center.

## Methods

### Data collection and study population

All consecutive patients treated with inner-branch custom-made and off-the-shelf aortic stent grafts with at least four branches by manufacturer Artivion® (Hechingen, Germany) between 01/2019 and 12/2022 were prospectively recorded in the Department of Visceral, Thoracic and Vascular Surgery at the Carl Gustav Carus University Hospital, Dresden. The data for each case was analyzed retrospectively based on electronic patient records and imaging. Demographics, comorbidities, radiologic data (anatomic features of the aneurysms, and target vessels), treatment modalities, complications, length of hospital stay, and follow-up examinations were collected. Exclusion criteria were patients with confirmed rupture and devices with less than four inner branches.

### Ethics approval

All procedures in studies involving human participants complied with the ethical standards of the institutional research committee.

Under the guidelines for research on human subjects, the local ethics committee at the Technische Universität Dresden approved the study (decision number BO-EK-87022023). The ethics committee was registered as an institutional review board (IRB) at the Office for Human Research Protections (OHRP) (registration number (IRB00001473 and IORG0001076).

### Treatment selection and procedure planning

The iBEVAR repair was offered to patients considered appropriate by the head of the department and the company and after careful assessment for open/endo repair possibilities. This decision was reviewed in all cases after a multidisciplinary vascular board and anesthesiologic assessment. Preoperative work-up included echocardiography and pulmonary function. In the last years, iBEVAR was increasingly favored over FEVAR or BEVAR due to one or more of the following indications:

1. Unfavorable target vessel angulation for FEVAR.
2. Narrow aortic lumen (<28 mm) at the visceral segment.
3. Missing circumferential contact with the aortic wall at the level of the fenestrations/branches.
4. Type Ia endoleak after failed endovascular aortic repair (EVAR) with a short or severely angulated neck (BEVAR in EVAR).

Endograft procedures were planned according to patient-specific anatomy using thin-slice computed tomography angiography (CTA) and multiplanar reconstructions. All CTA scan measurements were analyzed by an expert operator and compared with the ones obtained by the Artivion engineering team before final approval. All decisions were finally discussed with the patients, and ideally their relatives and alternative treatment options (i.e., open repair) were offered when suitable. Informed consent for the operation was obtained from all patients.

### Stent grafts design

**Figure 2:**
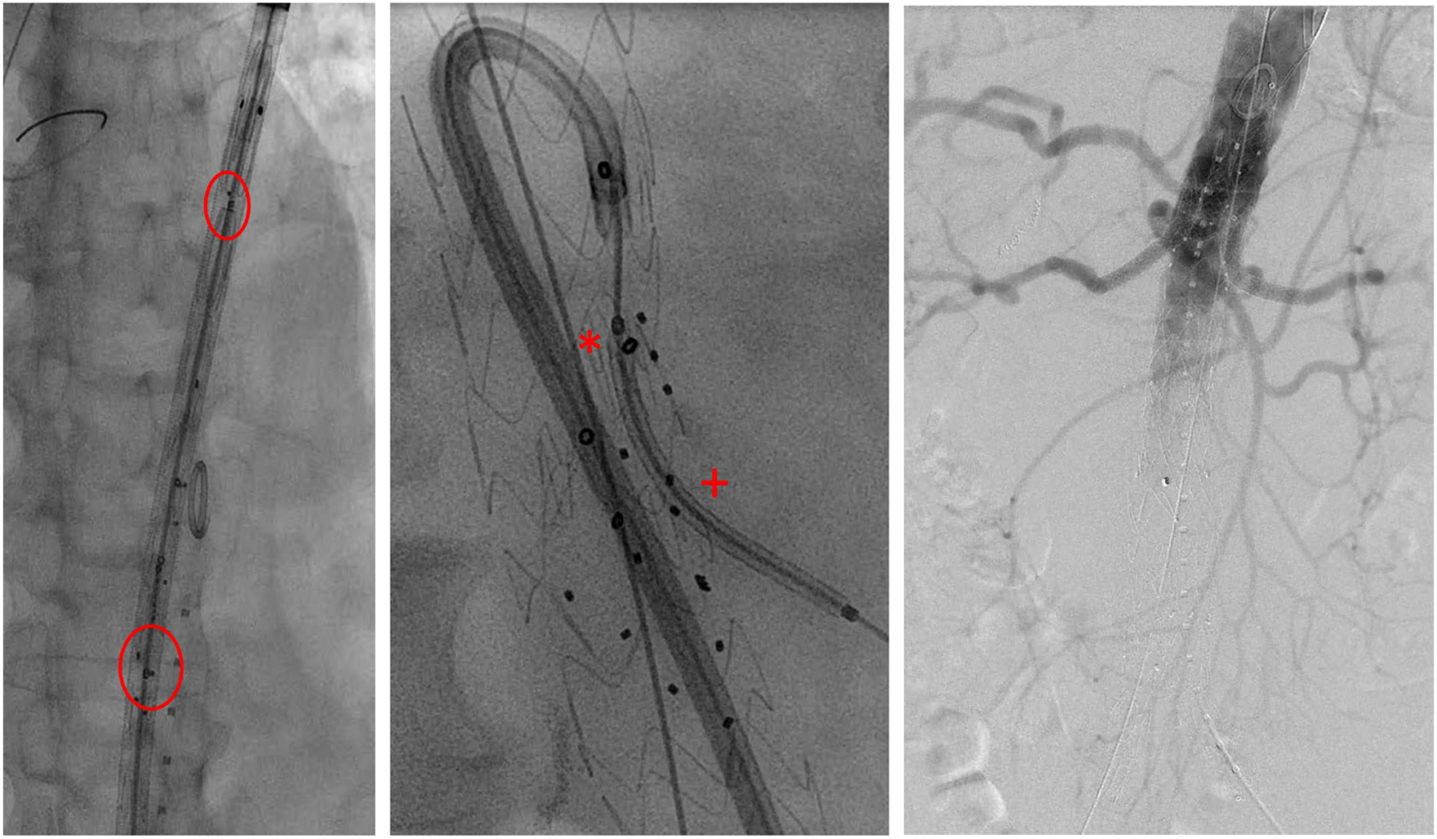
Intraoperative imaging. Left: stent graft depicted before deployment in fluoroscopy (circle: E-markers for orientation). Middle: cannulation of AMS-branch using a steerable sheath with bridging stent in position (*branch inlet marker; ^+^branch outlet marker). Right: final angiography.

The used endografts include inner-branch endografts, i.e., custom-made E-xtra Design and off-the-shelf E-nside stent grafts (Artivion, Hechingen, Germany). The inner branches were preferentially designed in an antegrade configuration with diameters of 8 and 6 mm for the coeliac trunk (CA) and superior mesenteric artery (SMA) and for the renal arteries, respectively. All branches have an enlarged and oval-shaped outlet to allow variability of the bridging stents. The augmentation of the branch outlets should allow the orientation of the bridging stent graft in many axial and sagittal directions, thus enabling the evolution from custom-made application to an off-the-shelf device, which has now been accomplished for the E-nside prosthesis (17). The endografts were loaded on a 24F delivery system. The rotational orientation of the endograft is based on appropriate visualization of the “E” markers of the device. A ring-shaped radiopaque marker is positioned at the inlet of each inner branch and three dot markers at their outlets, allowing orientation under fluoroscopy (Figure 2).

In the case of E-nside, the stent graft is available in four different versions with proximal diameters of 38 and 33 mm and distal diameters of 30 and 26 mm. All four inner branches are pre-canulated. Further specifications can be found in the Instructions for Use (IFU) (17).

### Procedure technique

All procedures were performed in a hybrid operating room under general anesthesia. Patients were administered systemic heparin to maintain activated clotting times (ACT) equaled to 250 s (checked in 30-min intervals). Lumbar drain for prevention of spinal cord ischemia was placed in selected cases at the discretion of the surgeon, generally in patients requiring long segment repair.

Ultrasound-guided percutaneous femoral access was obtained on both sides including closure devices. Additional open left axillary access was obtained where necessary. The endograft was deployed under fluoroscopy guidance with the markers of the inner-branch exit positioned 5 to 10 mm above the target vessel ostium. The rotational orientation of the endograft was based on the appropriate adjustment of the “E” markers.

Over time, a total femoral approach using a steerable sheath (Oscor, Florida, United States) was established whenever possible (Figure 2). Balloon-expandable stent grafts [primarily VBX stent graft (W.L. Gore & Associates, Flagstaff, AZ, United States), Advanta V12 (Maquet-Atrium Medical Inc., Hudson, NH, United States), or iCover (iVascular, Sant Vicenç dels Horts, Barcelona)] were used based on surgeon's choice and availability.

## Postoperative course

For spinal perfusion protection, a mean arterial pressure of >80 mm Hg was aimed in accordance with the current European Society for Vascular Surgery (ESVS) guidelines (18). The duration of lumbar drainage was 24–36 h. If no neurologic deficit occurred, the drain was clamped for an additional 6–8 h before removal.

Patients subsequently received ASA 100 mg and clopidogrel 75 mg for 6 months without loading doses. Thereafter, only aspirin was continued.

**Figure 3:**
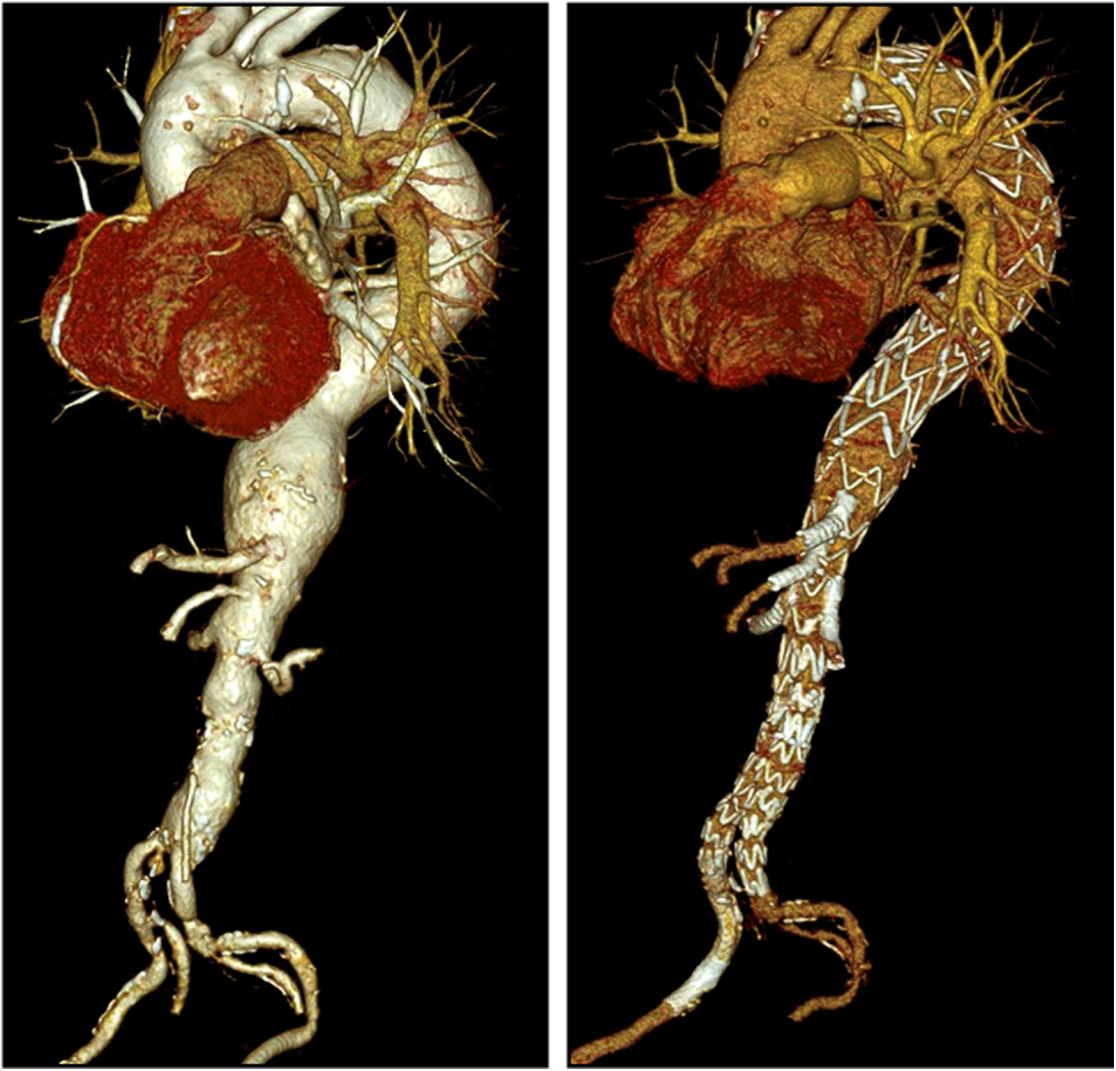
Pre- and postoperative CTA in 3D volume rendering technique. Left: preoperative TAAA type II. Right: postoperative after iBEVAR. CTA, computed tomography angiography; TAAA, thoracoabdominal aortic aneurysm.

CTA was performed on the first or second postoperative day (Figure 3). Routine follow-up consisted of clinical examination, duplex sonography, and CTA every 3–6 months during the first year and at least annually after that. All follow-up CTA studies were reviewed by a radiologist and analyzed within a vascular multidisciplinary board.

### Outcome parameters and definitions

The primary endpoint of this study was the technical and clinical success with morbidity and mortality rates in the perioperative period. According to the reporting standards for complex aortic repair by Oderich et al., clinical success was defined as successful deployment and implantation of the aortic modular components and side branches in addition to the absence of important disabling permanent clinical sequelae (e.g., death, aneurysm rupture, graft infection, conversion, paraplegia, and other major complications) (19). Secondary outcome parameters were overall survival, patency, and re-intervention rates during follow-up. Target vessel success was defined as successful cannulation and stent implantation in the target vessel without evidence of peripheral embolism or dissection and proper branch perfusion. The perioperative period was defined as the first 30 days after treatment or during a hospital stay if the length was more than 30 days. The maximum aortic diameter was assessed by computed tomography as the axial outer–outer diameter. The aortic diameter in the reno-visceral segment (IV) was measured at the level of the superior mesenteric artery. The target vessel diameter was determined in the first centimeters after vessel takeoff. All measurements were made after axial alignment in multiplanar reconstruction.

Complications were categorized according to the Society for Vascular Surgery (SVS) reporting standards for endovascular aortic repair and the Clavien–Dindo classification (19, 20). Technical success was defined as the correct placement of the main body and bridging stents and exclusion of the target pathology without evidence of type I or III endoleaks in accordance with the reporting standards for endovascular aortic aneurysm repair (9). Assisted primary success was defined if further unplanned treatment procedures (e.g., due to a type Ia endoleak) were necessary during the primary procedure for the exclusion of the target pathology. The follow-up period was the period from hospital discharge until the last available clinical examination.

### Statistical analysis

Statistical analysis was performed using IBM SPSS for Windows, Version 21.0 (IBM Corp., Armonk, NY, United States). All clinical characteristics were grouped to build categorical or nominal variables. Dichotomous variables were recorded as absolute frequencies (number of cases) and relative frequencies (percentages). Continuous data are presented as mean and SD, non-symmetrical with median, and interquartile range (IQR). Pearson's chi-squared or Fisher's exact test was used to analyze categorical variables. Differences between means were tested with a *t*-test or Mann–Whitney *U*-test. Survival and patency data were analyzed using Kaplan–Meier estimates, and differences were appointed by the log-rank test. A two-sided *P*-value <0.05 was considered statistically significant.

## Results

### Study population and patient characteristics

The study included 44 patients (81.8% male, age 76.57 ± 6.58 years). Since 2017, there has been a steady increase in the number of patients treated with iBEVAR. A total of 34 patients and 10 patients (22.7%) received a custom-made and an off-the-shelf prosthesis with four (90.9%; *n* = 40) or five inner branches (9.1%; *n* = 4), respectively, all downward facing. An endograft body with integrated iliac limbs was used in 11 patients, and completion EVAR (plus iliac branch device) followed in 19 (two) patients, respectively. Comorbidities and risk factors are shown in Table 1.

**Table 1:** Demographic and clinical data.

Variable^a^	*n* = 44 (%)
Demographic data	
Age (years)	76.57 ± 6.48
Sex (male/female)	36/8 (81.8/18.2)
Risk factors and comorbidities	
Chronic kidney disease^b^	11 (25)
Heart failure (>NYHA II)	10 (22.7)
Atrial fibrillation	8 (18.2)
Hypertension	44 (100)
CHD	14 (31.8)
Peripheral artery disease	12 (27.3)
Myocardial infarction	13 (9)
Diabetes mellitus	15 (34.1)
COPD	7 (15.9)
Nicotine abuse	19 (43.2)
Coincident aortic pathologies^c^	
Dissection	1 (2.3)
PAU	3 (6.8)
IMH	2 (4.5)
Aneurysm	12 (27.3)

CHD, coronary heart disease; COPD, chronic obstructive pulmonary disease; IMH, intramural hematoma; PAU, penetrating aortic ulcer.

^a^ Continuous data presented as mean ± SD.

^b^ GFR < 30 ml/min/1.73 m^2^.

^c^ Independent of the indication pathology.

### Indications

Treatment indications were TAAAs in 23, complex abdominal aneurysms in 11, and type Ia endoleaks (iBEVAR in EVAR) in 10 patients (Table 2). Thoracoabdominal pathologies included aneurysms (36.4%; *n* = 16), secondary expanded aortic dissections (6.8%; *n* = 3), and penetrating aortic ulcers (*n* = 3) and intramural hematoma (*n* = 1). The mean maximum aortic diameter was 63.9 ± 13.6 mm, and the mean aortic diameter in the reno-visceral segment (IV) was 25.5 ± 4.2 mm. Of note, despite rupture being an exclusion criterion, no iBEVAR for rupture was performed during the study period.

**Table 2:** Indications.

Variables^a^	*n* = 44 (%)
TAAA^b^	16 (36.4)
Type 2	5 (31.3)
Type 3	2 (12.5)
Type 4	8 (50)
Type 5	1 (6.3)
Distal descending/visceral aortic pathologies	7 (15.9)
PAU	3 (6.8)
IMH	1 (2.3)
Secondary expanding type B dissection	3 (6.8)
Complex AAA (para-/suprarenal)	11 (25)
Endoleak type IA from previous EVAR	10 (22.7)
Aortic diameters	
Minimum diameter segment IV (mm)	25.5 ± 4.3
Maximum diameter (mm)	63.9 ± 13.6

TAAA, thoracoabdominal aortic aneurysm IMH, intramural hematoma; PAU, penetrating aortic ulcer; AAA, abdominal aortic aneurysm.

^a^ Continuous data presented as mean ± SD.

^b^ According to the Crawford classification (19).

### Technical results

Lumbar drain was established in 12 patients pre-operatively (42% TAAA; 34% type 1a endoleak repair). An entirely percutaneous implantation was possible in 75% (*n* = 33) of the patients, and access *via* an iliac conduit was necessary in four patients (9.1%). Primary technical success after complete implantation was 95% (42/44). Two unexpected immediate-type Ia endoleaks were treated by proximal extension during the same session (primary assisted technical success 100%). The target vessel success was 99% (178/180) (Table 3). One dissection of the renal artery with consecutive occlusion with the subsequent need for nephrectomy was seen. In another patient, cannulation of the left renal artery proved to be frustrated despite all efforts. In both cases, the inner branch was successfully occluded with a vascular plug. The median contrast volume used was 200 ml (range, 85–350 ml) with a median fluoroscopy time of 85 min (range, 44–136 min). There were eight (18%) unplanned procedure extensions due to access complications (Table 4).

**Table 3:** Bridging stent grafts and target vessels in *n* = 44 patients.

		Celiac trunk	Superior mesenteric artery	Right renal artery	Left renal artery	∑
Stent type (%)	Viabahn VBX	11 (25)	12 (27)	15 (34)	11 (25)	49 (28)
	Advanta V12	24 (55)	24 (55)	19 (43)	23 (52)	90 (51)
	iCover	8 (18)	8 (18)	6 (14)	6 (14)	28 (16)
	Other	1 (2)	—	4 (9)	2 (5)	7 (4)
Additional lining (uncovered) (%)		3 (7)	1 (2)	5 (11)	4 (9)	13 (7)
Bridging stent extension (%)^b^		16 (36)	8 (18)	8 (18)	8 (18)	40 (23)
Target success (%)		44 (100)	44 (100)	44 (100)	42 (96)	178 (99)
Vessel angulation^a^ (°)		44.1 ± 20.7	41.7 ± 14.2	64.6 ± 23.5	67.3 ± 17.8	—
Vessel diameter^a^ (mm)		6.9 ± 1.4	8.2 ± 1.7	5.9 ± 0.9	5.9 ± 1.1	—

^a^ Continuous data presented as mean ± standard deviation.

^b^ Additional stent implantation necessary.

**Table 4:** Perioperative course and complications^a^ according to SVS reporting standards for endovascular aortic aneurysm repair (19).

Variables	*n* = 44 (%)
Intraoperative mortality	—
Primary technical success	42 (95.5)
Type Ia endoleak	2 (4.5)
Primary assisted technical success	44 (100)
Aortic dissection (within 30 days of AAA repair)	1 (2.3)
Grade 1: incidentally noted, asymptomatic	—
Grade 2: resolved with endovascular repair	1 (2.3)
Grade 3: open repair or fatal	—
Arterial perforation or rupture	2 (4.5)
Grade 1: spontaneous closure	—
Grade 2: stent graft or limited retroperitoneal iliac repair	1 (2.3)
Grade 3: laparotomy/thoracotomy	1 (2.3)
Access artery dissection or thrombosis	3 (6.8)
Grade 1: non-flow limiting dissection, local repair	2 (4.5)
Grade 2: stent, limited retroperitoneal bypass	1 (2.3)
Grade 3: conversion to open AAA repair	—
Access site false aneurysm	4 (9.1)
Grade 1: resolved spontaneously, compression/thrombin	2 (4.5)
Grade 2: surgical repair	2 (4.5)
Grade 3: ruptured	
Access site infection	3 (6.8)
Grade 1: resolved with oral antibiotics	—
Grade 2: operative drainage, intravenous antibiotics	2 (4.5)
Grade 3: major debridement, artery repair	1 (2.3)
Insufficiency closure system	4 (9.1)
Reno-visceral ischemia	2 (4.5)
Bowel resection	1 (2.3)
Nephrectomy	1 (2.3)
Acute limb ischemia	4 (9.1)
Balloon catheter thrombectomy/embolectomy	1 (2.3)
Thrombectomy/endarterectomy	2 (4.5)
Bypass graft	1 (2.3)

AAA, abdominal aortic aneurysm.

^a^ According to SVS reporting standards for endovascular aortic aneurysm repair (19, 33).

### Early results (perioperative)

There was no in-hospital mortality. Complete permanent SCI developed in 6.8% (*n* = 3) of patients immediately after the procedure. All these patients were treated due to a thoracoabdominal aneurysm (*n* = 2 type II, *n* = 1 type IV). No transient or late SCI was observed. One of these patients had already received a preoperative lumbar drain, the two other affected patients immediately after symptom onset. In addition, immediate postoperative lumbar drainage showed no symptom improvement in the two affected patients. Further neurological complications included one minor stroke on postoperative day 5. The combined morbidity was 45% according to the Clavien–Dindo classification (Tables 4, 5). In detail, access site complications occurred in seven (15.9%) patients (4× false aneurysm; 3× surgical site infection). Two (4.5%) and 10 (22.7%) patients developed relevant cardiac and pulmonary complications (2.3% re-intubation), respectively. Temporary dialysis was necessary for three (6.8%) patients. All patients were dismissed without dialysis.

**Table 5:** Systemic complications^a^ and grading^b^ according to Clavien–Dindo classification (20).

Variables	*n* = 44 (%)
Cardiac	
Grade 1: little or no hemodynamic consequence	3 (6.8)
Grade 2: symptomatic necessitating intravenous medication or PTCA	2 (4.5)
Grade 3: cardiac arrest, resuscitation	—
Pulmonary	
Grade 1: recovery with medical treatment	—
Grade 2: prolonged hospitalization/intravenous antibiotics	9 (20.5)
Grade 3: intubation, tracheostomy, deterioration in pulmonary function	1 (2.3)
Renal insufficiency	
Grade 1: no dialysis	4 (9.1)
Grade 2: temporary dialysis, prolonged hospitalization, permanently reduced renal function	3 (6.8)
Grade 3: permanent dialysis	—
Cerebrovascular	
Grade 1: temporary deficit with recovery within 24 h	3 (6.8)
Grade 2: delayed recovery, infarct on CT or magnetic resonance, permanent deficit with mild impairment	1 (2.3)
Grade 3: severe impairment or fatal outcome	—
Bowel ischemia	
Grade 1: recovered without intervention	—
Grade 2: recovered with intravenous antibiotics	—
Grade 3: bowel resection	2 (4.5)
Spinal cord ischemia	
Grade 1: resolution within 24 h	—
Grade 2: resolution within 1 month or minor permanent deficit, able to walk without support	—
Grade 3: major permanent deficit	3 (6.8)
Septic disease pattern	4 (9.1)
Clavien–Dindo grading complications^b^	20 (45.5)
IIIa	10 (22.7)
IIIb	7 (15.9)
IVa	3 (6.8)
IVb	—
In-hospital mortality	0 (0)

PTCA, percutaneous transluminal coronary angioplasty.

^a^ According to SVS reporting standards for endovascular aortic aneurysm repair (19).

^b^ According to the Clavien–Dindo classification (20).

Routine postoperative CTA revealed a type III endoleak in three patients (8.6%). These patients received a direct re-intervention with balloon dilatation/stent deployment at sealing zones. Furthermore, one patient showed an asymptomatic retrograde type B dissection, which was treated endovascularly 3 weeks later to allow possible spinal cord blood supply conditioning. No relevant stent migration or branch stenoses were observed. The mean hospital length of stay was 16 ± 19 days and 4 ± 11 days in ICU.

### Short- and midterm results (follow-up)

**Figure 4:**
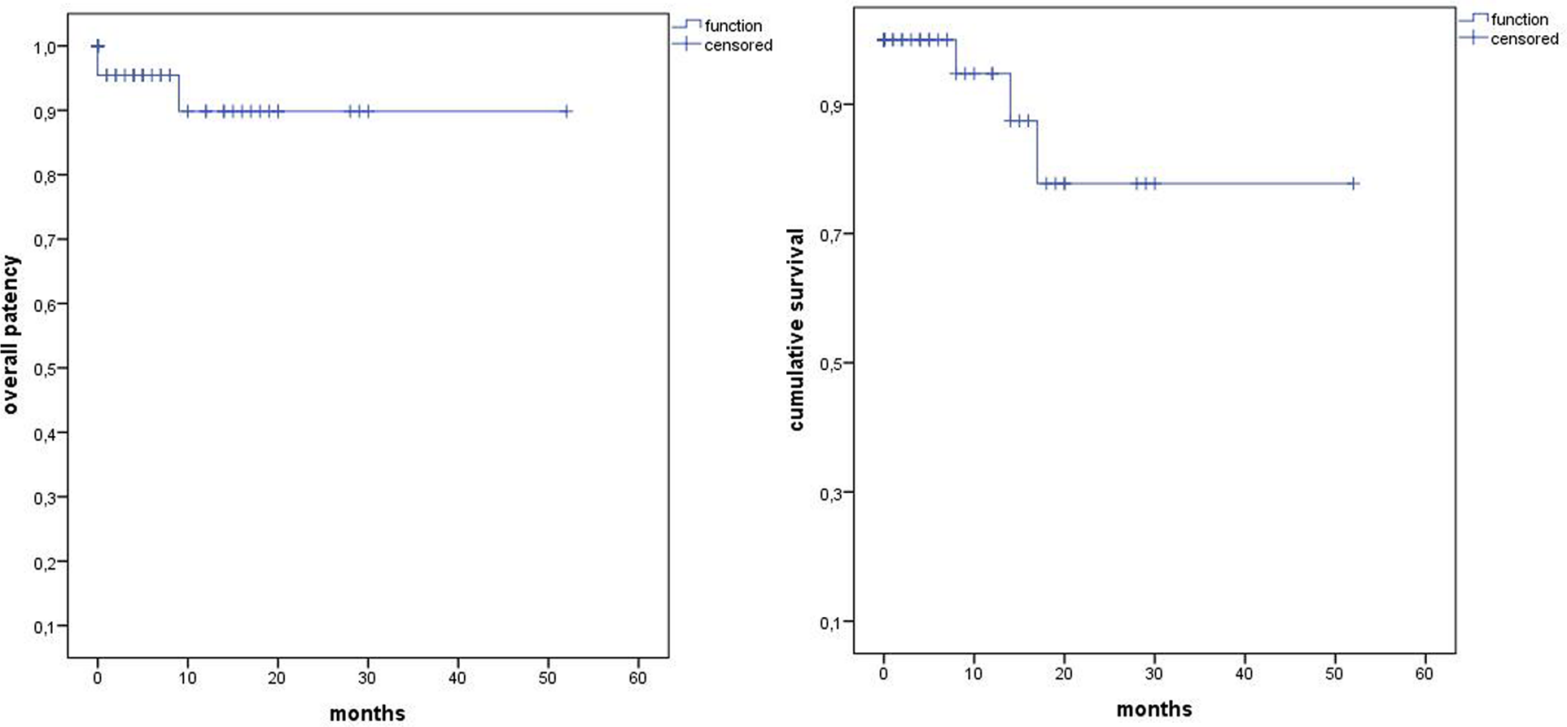
Kaplan–Meier estimates of overall survival and overall patency.

**Figure 5:**
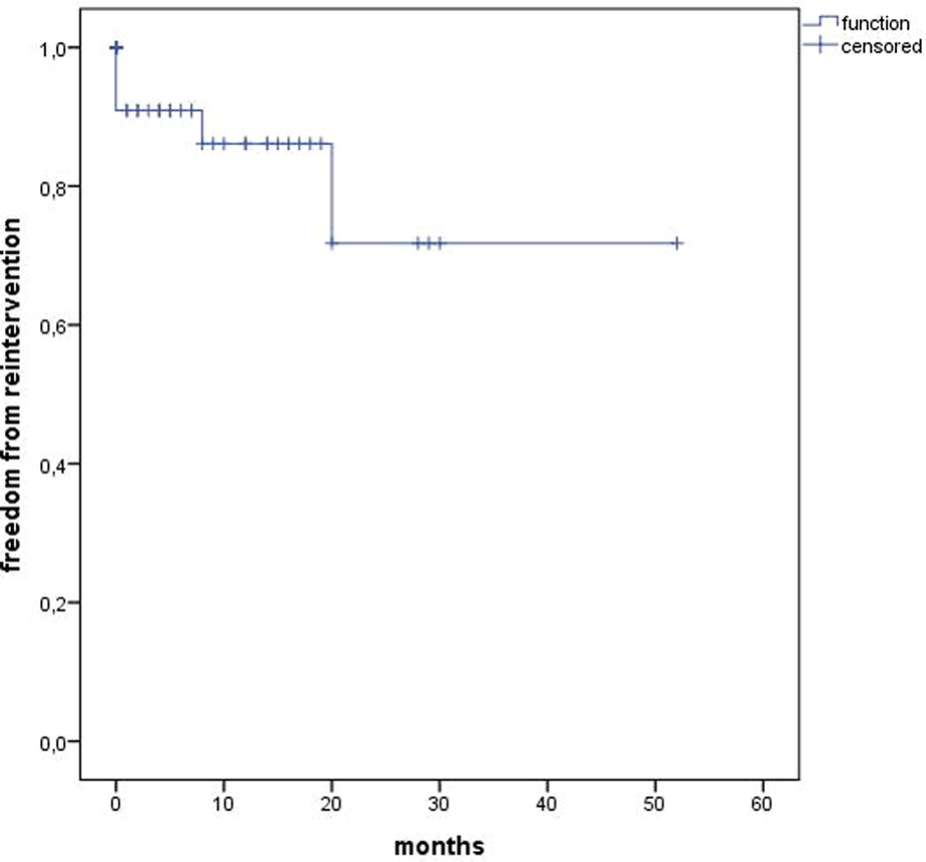
Kaplan–Meier estimates for freedom from re-intervention.

The median follow-up was 12 months (range 0–52). Estimated Kaplan–Meier 1-year survival manifested at 95% and branch patency at 98% (177/180) (Figure 4). One branch occlusion of the celiac trunk (asymptomatic, at 9 months) was seen. During follow-up, two relevant type 2 endoleaks were treated successfully in two patients [*n* = 1 coiling inferior mesenteric artery (20 mm progress in 18 months) and *n* = 1 polymer embolization of the aneurysm sac (9 mm progress in 9 months)]. Overall, re-intervention (due to aneurysm sac enlargement) was necessary for six patients including the three endoleaks treated during the same hospitalization (*n* = 5 endoleaks, *n* = 1 retrograde type B dissection) (Figure 5). Of three late deaths, one was aortic-related due to a stent graft infection.

## Discussion

This study demonstrates high technical success and comparable in-hospital, midterm, and long-term survival and re-intervention rates to classical fenestrated or outer-branched endografts for a mixed cohort of 44 patients treated with custom-made and off-the-shelf iBEVAR aortic prosthesis (11, 13, 15, 21, 22). This is currently one of the largest series on this relevant topic.

Although fenestrated and outer-branch aortic endografts have evolved to be applicable options for the treatment of thoracoabdominal aortic pathologies and complex AAAs, there are some specific limitations of the existing technology (11, 13, 21). So far, fenestrated endografts are preferred when aortic wall apposition is given at the origin of the visceral vessels. The fenestrations can be accessed through the femoral arteries, avoiding the need for an upper limb (brachial artery) access. But the sealing between the bridging stent and the main body relies only on the reinforced fenestration ring, comprising the risk for stent migration or fracture (14, 23, 24). In contrast, outer branches offer a stable overlap between the main body and the bridging stent. However, a wider aortic lumen is required (14). This was a relevant restriction, given the diameters seen in our cohort (Table 2).

Here, iBEVAR is a valid alternative (25–27). Historically, Abisi et al. reported that up to 16% of their patients were considered to be more suited for an iBEVAR due to severe angulation and narrow working lumen (11). Also, we were able to demonstrate a wide applicability for various aortic pathologies (Table 2).

Previous numerous reports on the outcome of F/BEVAR procedures showed high technical success with low perioperative mortality and morbidity but high re-intervention rates (8, 9, 22, 28). In a prospective multicenter study for fenestrated endovascular treatment of juxtarenal abdominal aortic aneurysms, Oderich et al. reported 100% technical success with no perioperative mortality and 22% secondary interventions (22). Doonan et al. observed a 30-day mortality of 6.3% and 5.7% of SCI, as well as 13.5% re-interventions in 141 patients with thoracoabdominal pathologies (28). A systematic review and meta-analysis for the t-Branch off-the-shelf endograft (197 patients, 19% urgent) determined a pooled technical success of 92.8%, 5.8% early mortality, and 12.2% spinal cord ischemia (8).

To date, there are only a few studies that reported results on inner-branch endografts for the visceral segment, and most of them are confounded by various endograft configurations with fenestrations and outer branches (10–12, 29, 30). Silverberg et al. reported on 27 patients treated with inner-branch custom-made device (CMD) (90 inner branches) with high technical success (96%) and low complication rates (3.7% (*n* = 1) in-hospital mortality and spinal cord ischemia, respectively) (13). Abisi et al. observed no major complication or 30-day mortality in 18 patients provided with CMD inner branch (11). So far, the largest multicenter study from Italy by Simonte et al. reports on 45 patients treated with a CMD inner-branch graft with reasonable technical success (93.3%), no in-hospital mortality, and 6.7% (*n* = 3) persistent spinal cord ischemia. They publicized internal data provided by Artivion, that the request for inner branches increased up to 10-fold, and described inner branches as their preferred treatment device (14).

In contrast to this, Katsargyris et al. described the catheterization of inner branches as a difficult procedure in visualizing and orientating to identify the inlet of the branch (12). Based on our experience and with a corresponding learning curve, we were recently able to perform 75% of the procedures exclusively percutaneously from transfemoral *via* a steerable sheath enabling the reduction of procedural steps and upper limb access complications (11, 31). In our experience, the working space in the main body was sufficient for cannulation (11). In comparison, our fluoroscopy time and amount of contrast volume were quite similar to previous publications (13, 14).

Considerable re-intervention rates are the Achilles heel of endovascular repair, increasing with the procedural complexity (7, 13–15, 23, 32). We observed an overall re-intervention rate due to endoleaks of 11.3% (*n* = 3), comparable with the previous literature (7, 10, 13, 14). It should be noted that the follow-up is still limited (mean follow-up of 12 months in this study) because the new implementation of this technology and long-term results are still pending. However, a direct comparison of re-intervention rates between FEVAR and outer-branch BEVAR is not appropriate, as most publications have used both technologies together without further distinction. Regardless, a recent study evaluated that branch endoleaks have a high rate (up to two-thirds) of spontaneous resolution and might resolve more often spontaneously compared with fenestration endoleaks. Further, they concluded that small target vessel endoleaks in pre-dismissal imaging may be initially observed and persistent or late endoleaks can be successfully treated by endovascular re-intervention (7).

By now, the E-nside grafts allow treatment with the advantages of inner branches in an off-the-shelf device. Demonstrated in a meta-analysis on the t-Branch, representing a widely accepted off-the-shelf solution for urgent/emergency treatments, acceptable clinical results with a mortality of 5.8% and 12.2% rate of spinal cord ischemia (1.2% permanent paraplegia) in elective and urgent cases can be reached (8).

There are several reports of access complications, which also appeared in our procedures (Table 4) (10, 13). Hence, careful case planning of the access is crucial to reduce complications.

This study has some limitations. First, it is limited by the small number of cases and to the retrospective non-randomized single-center study design, generating bias linked to a retrospective data collection and device selection. Furthermore, during the study period of 5 years, there has been a learning progress and gain in expertise with this endovascular technique that may have affected treatment procedures. Lastly, for teaching purposes, procedures might not be directly comparable due to confounding bias between operators.

## Conclusion

This retrospective study demonstrates that inner-branch endografts for complex aortic repair are a viable option, especially for narrow aortic visceral segment pathologies. Our results show excellent technical results and early outcomes with comparable and acceptable re-intervention and spinal cord ischemia rates. These encouraging results in a mixed cohort and elective and urgent setting may suggest iBEVAR as a future primary treatment for complex aortic pathologies warranting long-term results.

## Data Availability

The original contributions presented in the study are included in the article, further inquiries can be directed to the corresponding author.
